# A comprehensive suite of earthquake catalogues for the 2016-2017 Central Italy seismic sequence

**DOI:** 10.1038/s41597-022-01827-z

**Published:** 2022-11-18

**Authors:** Lauro Chiaraluce, Maddalena Michele, Felix Waldhauser, Yen Joe Tan, Marcus Herrmann, Daniele Spallarossa, Gregory C. Beroza, Marco Cattaneo, Claudio Chiarabba, Pasquale De Gori, Raffaele Di Stefano, William Ellsworth, Ian Main, Simone Mancini, Lucia Margheriti, Warner Marzocchi, Men-Andrin Meier, Davide Scafidi, David Schaff, Margarita Segou

**Affiliations:** 1 Istituto Nazionale di Geofisica e Vulcanologia – Osservatorio Nazionale Terremoti Via di Vigna Murata, 605 – 00143 Rome, Italy; 2 Lamont-Doherty Earth Observatory, Columbia University - 61 Rte. 9 W, Palisades, NY 10964 USA; 3 The Chinese University of Hong Kong - Shatin, NT, Hong Kong SAR, The People’s Republic of China; 4 Università degli Studi di Napoli ‘Federico II’ - Via Cinthia, 21, 80126 Napoli, Italy; 5 Università degli Studi di Genova, Dipartimento di Scienze della Terra, dell’Ambiente e della Vita - Corso Europa 26, 16132 Genova, Italy; 6 Department of Geophysics, 397 Panama Mall, Stanford University, Stanford, CA 94305-2215 USA; 7 University of Edinburgh, School of Geosciences, Grant Institute, James Hutton Road, Edinburgh, EH9 3FE UK; 8 Scuola Superiore Meridionale - Largo S. Marcellino, 10, 80138 Napoli, Italy; 9 ETH, Swiss Federal Institute of Technology - Rämistrasse 101, 8092 Zürich, Switzerland; 10 British Geological Survey, The Lyell Center, Research Avenue South, EH14 4AP Edinburgh, UK

**Keywords:** Seismology, Natural hazards

## Abstract

The protracted nature of the 2016-2017 central Italy seismic sequence, with multiple damaging earthquakes spaced over months, presented serious challenges for the duty seismologists and emergency managers as they assimilated the growing sequence to advise the local population. Uncertainty concerning where and when it was safe to occupy vulnerable structures highlighted the need for timely delivery of scientifically based understanding of the evolving hazard and risk. Seismic hazard assessment during complex sequences depends critically on up-to-date earthquake catalogues—i.e., data on locations, magnitudes, and activity of earthquakes—to characterize the ongoing seismicity and fuel earthquake forecasting models. Here we document six earthquake catalogues of this sequence that were developed using a variety of methods. The catalogues possess different levels of resolution and completeness resulting from progressive enhancements in the data availability, detection sensitivity, and hypocentral location accuracy. The catalogues range from real-time to advanced machine-learning procedures and highlight both the promises as well as the challenges of implementing advanced workflows in an operational environment.

## Abstract (summary)

**Table:** 

Measurement(s)	Earthquakes location and magnitude
Technology Type(s)	Earthquakes location codes
Factor Type(s)	Hypocenters
Sample Characteristic - Environment	tectonic earthquake
Sample Characteristic - Location	Italy

## Background & Summary

National building codes prescribing earthquake-resistant design remain the backbone of earthquake risk reduction as they consider the seismic hazard of strong ground motions experienced over decades to centuries. But during a seismic sequence, the seismic hazard can fluctuate significantly from day-to-day, which may drive alternative mitigation actions such as closure of vulnerable buildings, emergency shoring up of others to relocation of populations from hazardous areas. Such measures are based on a scientific understanding of earthquake generation, e.g., its statistical behaviour or underlying physical processes. Advancing this understanding requires a continuous improvement of sequence-specific information in near real-time. The earthquake catalogue is the primary tool, and its content depends on the underlying observational methodologies. Recent advances in machine learning applied to earthquake detection and characterization currently boost the information content of catalogues by significantly lowering the detection threshold and include more small-magnitude events. Advanced workflows for improved location accuracy provide sharper resolution of structures that have great potential for gaining new insights into the underlying processes.

**Fig. 1:**
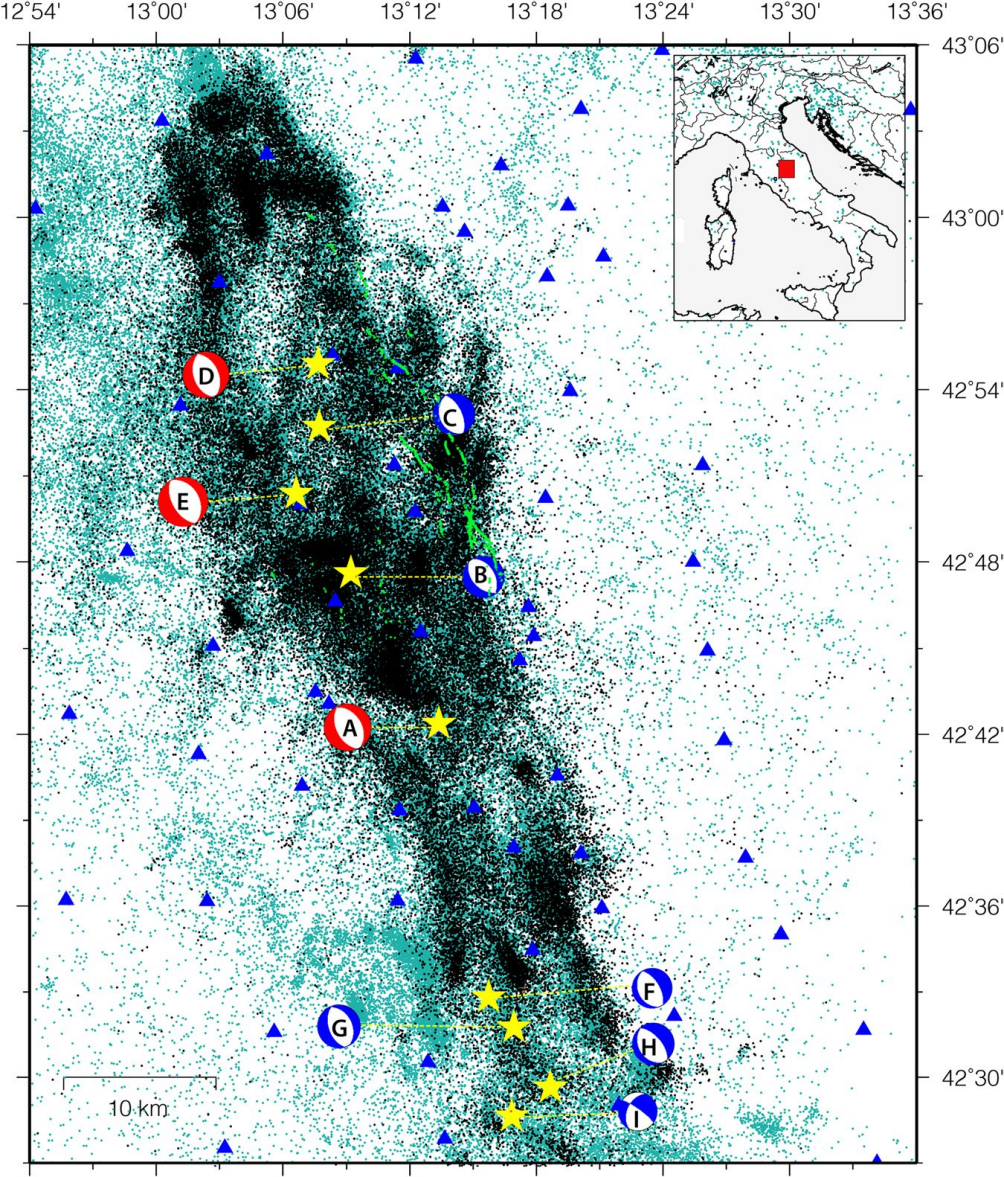
Map of the study area. Green points refer to events that occurred between 1981 and 2016 before the sequence onset (Chiaraluce and Di Stefano, p.c.), whereas the black-coloured events occurred during the sequence, between 23 August 2016 and 31 August 2017 as contained in the CAT0 catalogue (i.e., 73,009 events detected and recorded by INGV’s monitoring room). Yellow stars marked events with 5.0 ≤ Mw ≤ 6.5 with their focal mechanisms (from A to I) shown; red beach balls indicate the mainshocks with M_W_≥6.0 and blue ones with M_W_ < 6.0. The blue triangles denote seismic stations located within the map area while surface ruptures^25^ are reported as green lines.

The 2016–2017 central Italy sequence provides an opportunity to demonstrate the evolution of our observational capability and earthquake analysis methods. The sequence contained three main events with moment magnitudes *M*_w_ ≥ 5.9 and four *M*_w_5.0*‒*5.5 (Fig. 1). Together, they ruptured an 80-km long fault system of the central Apennines over a period of six months. This protracted sequence highlights the scientific challenge to track the evolution of a seismic sequence with multiple mainshocks and societal challenge to rapidly identify and characterize the evolving hazard.

The 2016–2017 central Italy sequence was recorded by a dense network of up to 155 seismic stations for over one year, owing to the rapid response effort of an Italian–UK scientific collaboration^1^ (Fig. 1). This collaboration resulted in the development of six high-quality earthquake catalogues, each derived using different approaches reflecting different operational and scientific requirements (i.e., ranging from robust real-time surveillance system to offline state-of-the-art methods). Most of this collection is the result of the NSFGEO-NERC project “*The central Apennines earthquake cascade under a new microscope*” (NE/R0000794/1), which investigated the complexity of earthquake interactions and developed physics-based and stochastic models to forecast the evolution of seismicity in space and time. While each of the catalogues has been described and the results interpreted in detail in separate publications, the goal here is to provide a comparative description of, and access to, all the catalogues together for subsequent analysis by the wider community. High-resolution earthquake catalogues have in fact the potential to provide more robust descriptions of the evolving sequence in several ways including illumination of previously undetected seismogenic faults^2^. Such structures are commonly underreported in real-time earthquake catalogues. We expect that these catalogues will motivate new analyses bringing new understanding of both the statistical nature of earthquake interactions and the underlying physics. Application of advanced workflows in other areas have revealed hundreds of thousands of hidden earthquakes^3,4^, providing new insights to hidden structures and the tectonic environment.

Current methods for time-dependent earthquake forecasts reside in a low-probability and high-uncertainty environment, which limits their operational use^5^. For instance, before the Central Italy sequence started with the *M*_W_6.0 Amatrice event, the probability that one or more *M* ≥ 4 earthquakes occur within the next week inside the area shown in Fig. 1 was ~0.8% (Marzocchi *et al*.^6^); any specific decision based on such numbers is not warranted^7,8^. As outlined in the following, the six catalogues presented here may have an impact on earthquake predictability research^9^, which could improve decision support during seismic sequences^10^.

The catalogues are facilitating the development of innovative forecast models^11,12^ to support better decision making during seismic sequences. The catalogues vary in their content and accuracy due to operational constraints and choices regarding event detection and association, location resolution, estimation of event magnitude and other source parameters. Most comprehensive catalogues are currently not available in near-real-time, but their potential short-term forecasting skill needs to be investigated and quantified. Attributes that increase forecast skill are promising targets for incorporating in operational workflows. Some advances such as near real-time relocation procedures (e.g., DDRT^13^) and machine-learning picker PhaseNet^14^ have already been adopted for operational monitoring in tectonic (Northern and Central California^13^) and volcanic (Axial Seamount^15^; Mayotte and Martinique islands^16^) areas. Specifically, the comprehensive catalogues will permit a more detailed examination of the magnitude–frequency distribution (MFD) as they extend to lower magnitudes. For instance, testing whether the Gutenberg–Richter (GR) relation holds at low magnitude (M_L_ < 1.5) is of paramount importance for understanding if b-value variations (i.e., the changing slope of the GR relation) have a physical meaning or if they result from departures from an exponential MFD^17,18^. These catalogues can help test hypothesis such as the predictive value of a spatiotemporal variations in terms of b-value (e.g., Gulia and Wiemer^19^; García-Hernández *et al*.^20^; Herrmann *et al*.^21^). With these catalogues, there are many more properties about earthquake occurrence that can be studied in more detail^22^, such as earthquake triggering, interaction, and spatiotemporal clustering.

## Methods

We describe here the set of six earthquake catalogues by providing necessary information on the procedures and techniques adopted to generate them. All the catalogues cover one year of seismic activity of the 2016‒2017 central Italy sequence. Activity initiated abruptly and without foreshocks on August 24 with a *M*_W_ 6.0 event (event A in Fig. 1; Tinti *et al*.^23^) near the town of Amatrice. A month later, it was followed on October 26 by the *M*_*W*_ 5.9 event near Visso (event D in Fig. 1). Four days later, on October 30, the largest event with *M*_W_ 6.5 occurred near the town of Norcia (event E in Fig. 1; Chiaraluce *et al*.^24^). This earthquake ruptured the entire length of the Mt. Bove and Mt. Vettore fault zone between the towns of Amatrice and Visso, including segments of the fault that slipped during the previous events as evidenced by surface ruptures^25^ (Fig. 1), coseismic slip models^26^ and aftershock distribution^27^. The sequence strengthened a final time on January 18, 2020, with a series of four events with 5.0 ≤ M_W_ ≤ 5.5 (events F, G, H, I in Fig. 1), that activated the southernmost segment of the fault system near Campotosto. Other notable events include a M_W_5.3 earthquake (B in Fig. 1) that occurred 1 hour after the Amatrice mainshock on an antithetic fault^24^, and a M_W_5.4 earthquake (event C in Fig. 1) that preceded the Visso event by 2 hours.

The catalogue set ranges from a standard routine catalogue generated by the real-time monitoring system at the Istituto Nazionale di Geofisica e Vulcanologia – INGV (CAT0^28^) to high-resolution catalogues generated offline with up-to-date standard (CAT4^29^) and machine-learning (CAT5^30^) approaches.

Real-time and derived conventional catalogues (e.g., CAT0 and CAT1) rely on a routine detection, visual inspection, and manual travel time measurements by an analyst. Consequently, such catalogues generally underreport small events because their focus is on properly capturing and characterizing the larger events. They also have a relatively low hypocentral location accuracy due to use of regional Earth models and single event location procedures. These limitations can result in poor spatial resolution of seismicity creating a vague depiction of the fault system. Yet, these preliminary catalogues typically include all major events (here above ~M_L_3.5)—including those found in the coda wave train of the largest events, when automatic approaches may miss many events—rendering these catalogues critical for assessing the stability of alternative catalogues. Creating a high-resolution earthquake catalogue in real-time during a seismic sequence is particularly difficult due to both the need of a series of cross check on the results and the increasing number of deployed seismometers (mainly in the first few days-weeks), which leads to variable network geometry and growing data volume.

### The earthquake catalogues

**Fig. 2:**
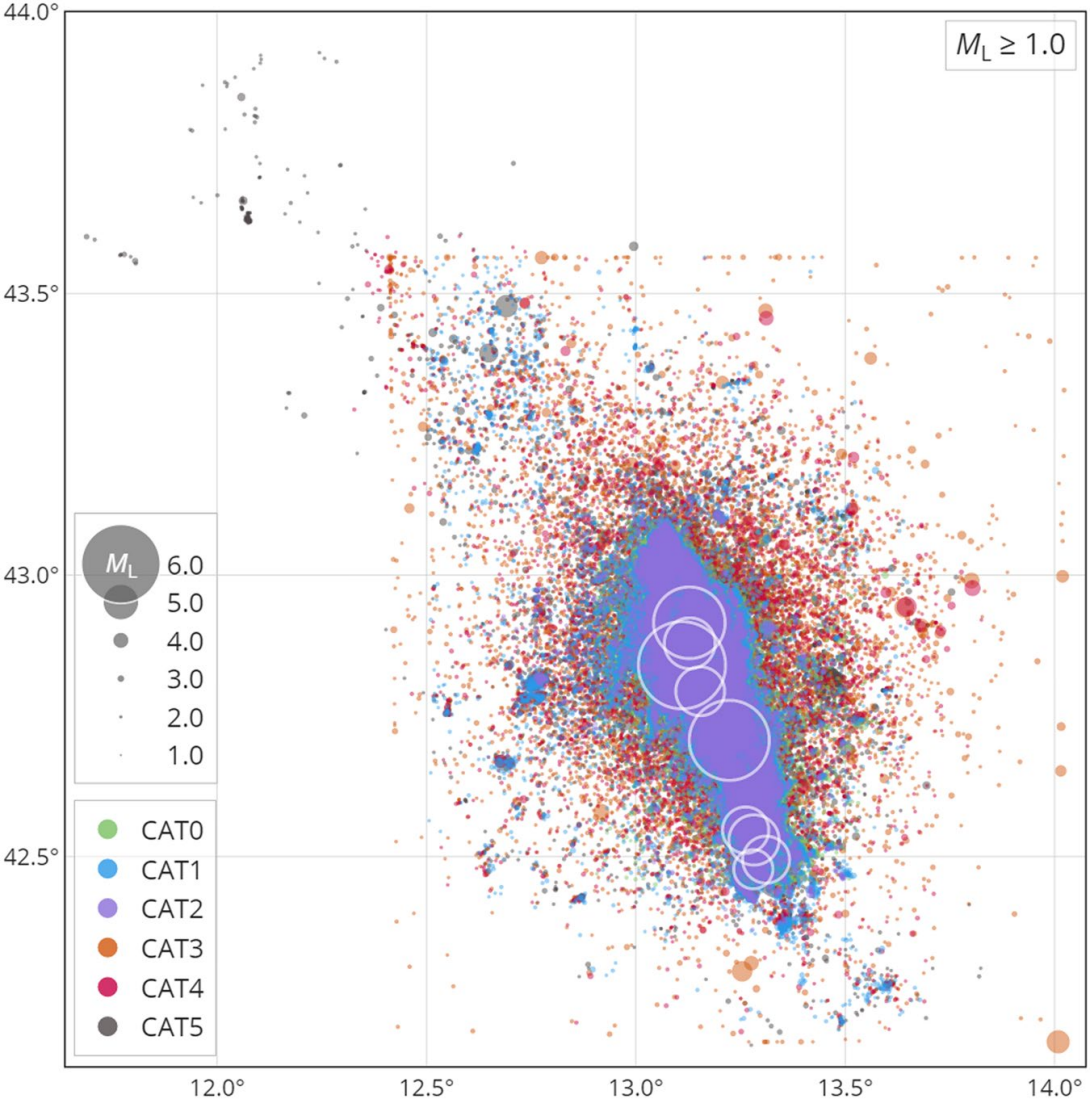
Spatial distribution of epicenters for the six catalogues, each represented by a separate colour (see legend), only for events with a local magnitude M_L_ ≥ 1.0. The white circles correspond to the larger events identified with stars in Fig. 1. Note that the circle sizes scale continuously with magnitude; the items in the legend only represent the sizes for integer values.

**Fig. 3:**
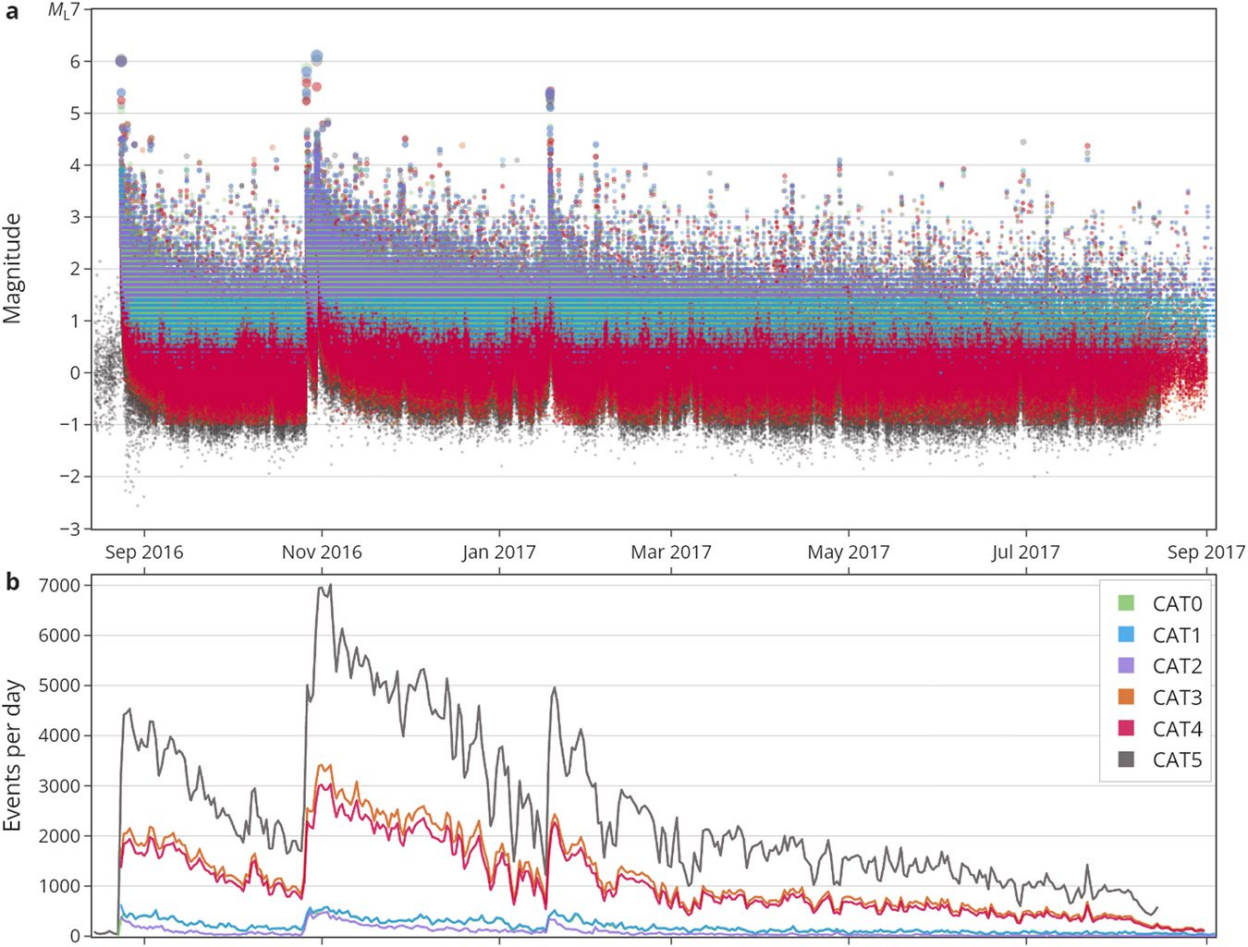
Timeline of event magnitudes (**a**) and event rates (**b**) of the six catalogues. Note that CAT0 is barely discernible and mostly overlaid by CAT1, which inherited CAT0’s events; the same applies to CAT3 and CAT4.

**Fig. 4:**
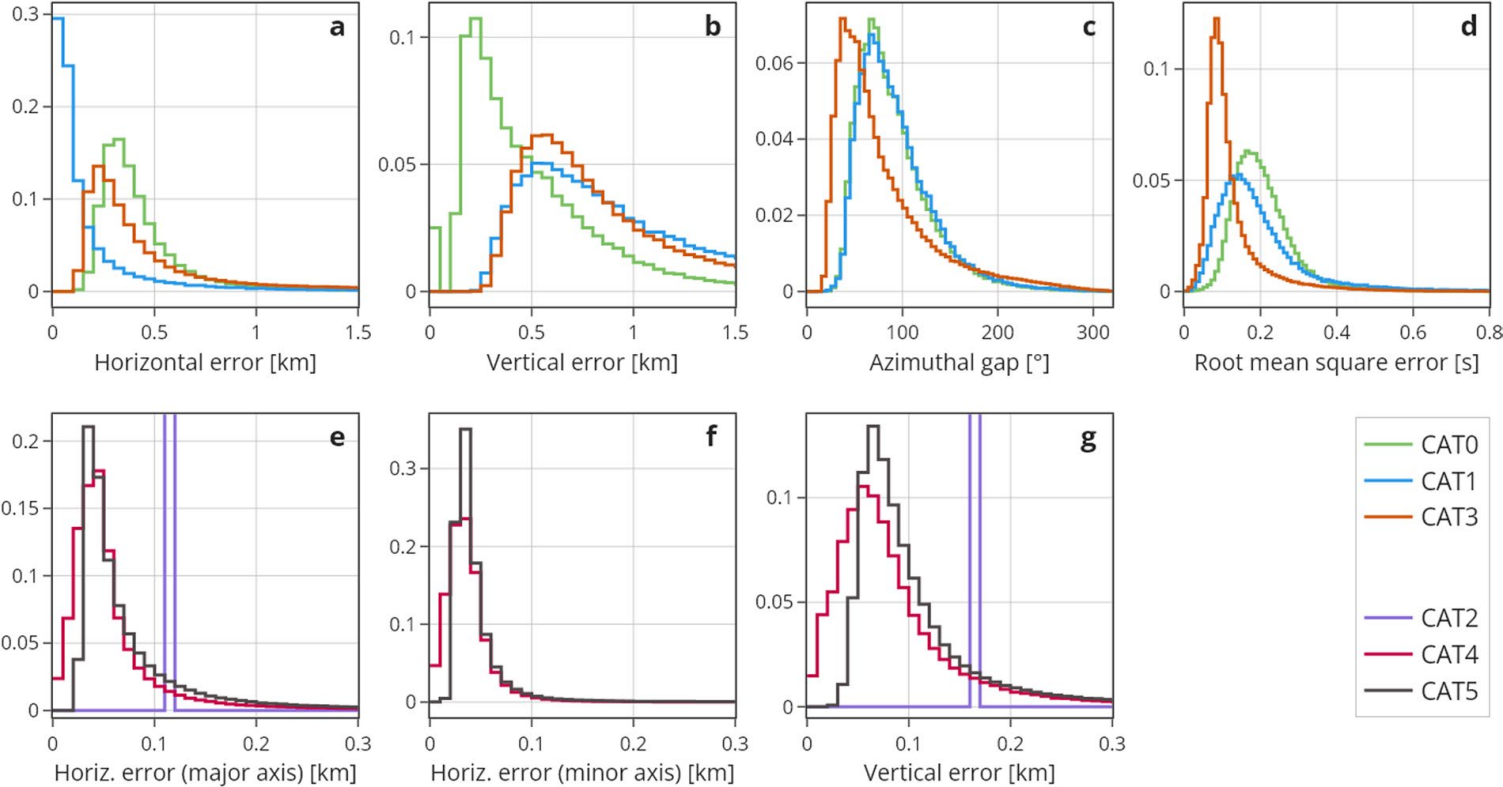
Normalized distributions of location uncertainties and quality parameters. Top row shows for CAT0, CAT1, and CAT3: absolute location errors in horizontal (**a**) and vertical direction (**b**), azimuthal gap (**c**), and root mean square error (**d**). Note that the location errors of CAT0 were derived differently from CAT1 and CAT3 and are overly optimistic. Bottom row shows for CAT4 and CAT5 the bootstrap relative location errors at the 95% confidence in horizontal direction for the major (**e**) and minor axis (**f**) of the error ellipsoid, and in vertical direction (**g**). For CAT2, only the average value of the horizontal and vertical location errors for a representative subset of the events are reported; these Dirac-delta-like distributions were added to the bottom row subfigures (**e** and **g**), because error estimation in CAT2 is most similar to CAT4 and CAT5.

**Fig. 5:**
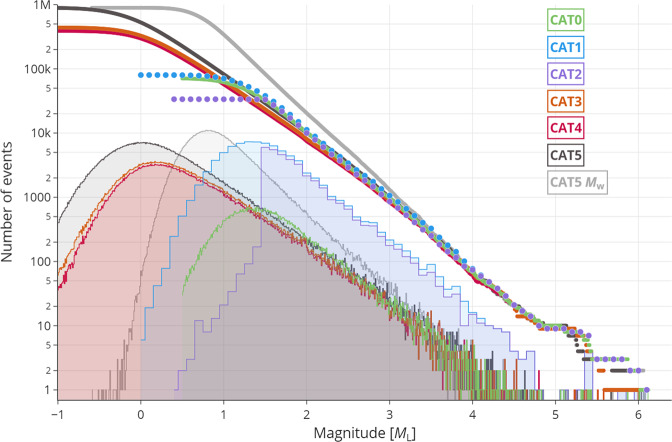
Magnitude–frequency distribution (MFD) of the six catalogues in terms of histogram (filled areas) and cumulative distribution (solid and dotted curves) for local magnitude, M_L_. Note that CAT1 and CAT2 have a 0.1 magnitude binning as opposed to the 0.01 magnitude binning of the other CATs (and therefore a coarser-stepped histogram and cumulative distribution). For CAT5, also MFD of the moment magnitude, M_w_, is shown (grey). The MFDs are truncated at M_L_−1.0.

**Fig. 6:**
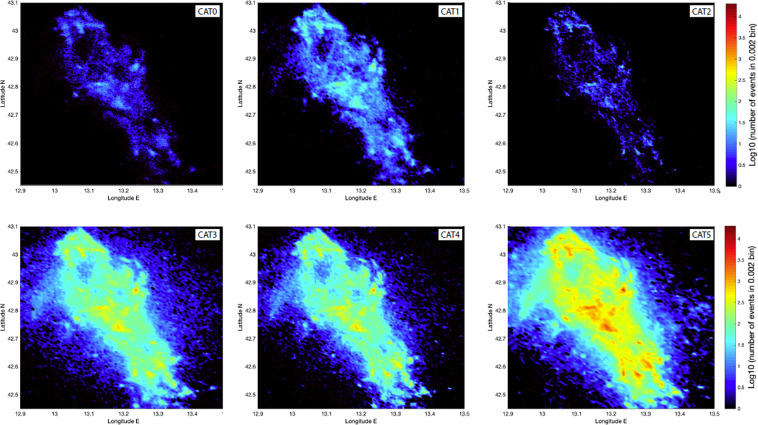
Maps showing the event density of each catalogue reported as Log10 of the number of events in 0.002 × 0.002 degrees (°) cells.

All six catalogues cover the period between August 2016 and August 2017. The attributes of all the catalogues are summarized in Table 2. Their properties  are compared qualitatively and quantitatively in terms of the spatial distribution of locations (Fig. 2), temporal evolution (Fig. 3), hypocentral locations quality parameters (Fig. 4), magnitudes, in terms of MFDs (Fig. 5), and spatial density (Fig. 6). Table 1 reports their time span, number of events, type of analysis, completeness magnitudes, and number of events above M_L_ > 4.

**Table 1:** Summary information for the six catalogues.

Name	Starting Date	Ending Date	Number of Events	Analysis	M_C_^MAXC^	M_C_^Lilliefors^	M_L_ > 4 Events
CAT0	23 August 2016	31 August 2017	73,009	RT	1.6	1.68	68
CAT1	24 August 2016	17 January 2018	82,356	NRT	1.5	2.80	77
CAT2	24 August 2016	17 January 2018	33,869	NRT	1.7	2.40	74
CAT3	24 August 2016	31 August 2017	440,727	OFL	0.4	2.52	70
CAT4	24 August 2016	31 August 2017	390,336	OFL	0.4	2.53	62
CAT5	15 August 2016	15 August 2017	900,058	OFL	0.2 (Mw: 1.0)	2.56 (Mw: 1.71)	64

M_C_^MAXC^ represents the magnitude of completeness computed with the maximum-curvature method^58^ and a + 0.2 correction^59^, whereas M_C_^Lilliefors^ is based on the Lilliefors test for an exponential MFD^57^.

The offline catalogues created using advanced event detection, seismic phase picking, and association algorithms and/or machine learning approaches, provide many more (six to ten times, see Table 1, Figs. 3 and 5) events and greater accuracy in the arrival-time measurements, allowing better quality of locations (Fig. 4, top right). In addition, multiple-event location techniques complemented by waveform cross-correlation measurements, lead to a significant improvement in the spatial resolution (Fig. 4), extending the reach of observational geology deep into the subsurface Table 2.

**Table 2:** Comparison of all the catalogues’ headers in different categories.

Category	CAT0	CAT1	CAT2	CAT3	CAT4	CAT5
Events Identification code	Id1	Id1	Id1	Id1		
				Id3		
				Id4	Id4	
						Id5
Origin time	Date	Date	Date	Date	Date	Date
	Time	Time	Time	Time	Time	Time
Location	Lat	Lat	Lat	Lat	Lat	Lat
	Lon	Lon	Lon	Lon	Lon	Lon
	Depth	Depth	Depth	Depth	Depth	Depth
Location parameter and quality	Errh	Errh	Errh	Errh		
	Errv	Errv	Errv	Errv		
	Gap	Gap	Gap	Gap		
	Rms	Rms	Rms	Rms		
	Nphs	Nphs	Nphs	Nphs		
					EH1	EH1
					EH2	EH2
					EZ	EZ
					AZ	AZ
				Qual		
				Class		
Magnitudes				ML_s	ML_s	
				Std_ML_s		
	Mpi	Mpi	Mpi	Mpi		
	ML	ML	ML			
	MW	MW	MW			
	MD	MD	MD			
	ML-MED					
		MW-M	MW-M	MW-M	MW-M	
						ML-N
						ML-mean
						ML-median
						Std-ML
						MW-REGRE
Focal mechanism solution		Strike	Strike	Strike		
		Dip	Dip	Dip		
		Rake	Rake	Rake		
Miscellaneous						Split

#### CAT0

This is the only catalogue of the 2016–17 sequence generated in real time. It consists of 73,009 events covering the period from 2016-08-23 to 2017-08-31 with INGV local magnitude^28^ M_L_ ranging 0.50 ≤ M_L_ ≤ 6.12. The earthquakes are detected and located by the INGV national seismic permanent network and monitoring room, connected to the Italian Civil Protection. P- and S-waves arrival times revised in nearly real-time (within 30 minutes) by the duty seismologists in the INGV seismic monitoring room are used to compute locations using a linearized inversion approach encoded in the IpoP code^31,32^. Travel times are computed using a coarse regional (nationwide) velocity model consisting of homogeneous 1D horizontal layers with fixed V_P_/V_S_ ratio (1.73^33^). Each event is independently located by analysts (seismologists) applying different setups in terms of starting location or readings and outliers’ removal with distance depending on the purpose. Thus, during a seismic crisis standard catalogues usually under-report small magnitude events (see Fig. 5). All events, however, are visually inspected and verified. They contain all the larger events of the sequence including most of the ones detectable in the coda of the mainshocks, usually missing in the automatically generated catalogues.

#### CAT1

This catalogue consisting of 82,356 absolute locations, is the extended version of the catalogue released by Chiaraluce *et al*.^24^. It covers the period from 2016-08-24 to 2018-01-17 with INGV local magnitude ranging 0.0 ≤ M_L_ ≤ 6.12. CAT1 was generated starting from the same the P- and S-wave arrival times of CAT0 with the addition of arrivals derived from 24 temporary stations deployed after the sequence onset. Hypocentral locations were determined using a layered 1D P- and S-wave velocity model with gradients. The model is a version of the layered minimum 1D model estimated for the region by Carannante *et al*.^34^. Hypocenters were determined using NonLinLoc^35^ with station corrections defined for the permanent seismic stations used in CAT0. These methods result in improved resolution of hypocentral locations reducing the mean location uncertainty for most of the events (about 60%) to about 300 m in latitude and longitude up to 600 m in depth (Fig. 4).

#### CAT2

This catalogue of relative locations by Michele *et al*.^27^ covers the period from 2016-08-24 to 2018-01-17 and includes all the 33,869 events with M_L_ ≥ 1.5 from CAT1. It also uses the the same velocity model and arrival times as CAT1. Hypocenters were located with the double-difference algorithm HypoDD^36^ with phase delay times measured using waveform cross correlation (e.g., Schaff *et al*.^37^). By inverting both absolute and relative arrival times, the spatial resolution of the 33,869 events was significantly improved with respect to CAT0 and CAT1. Formal errors, computed from the full covariance matrix using Singular Value Decomposition (SVD; see Waldhauser & Ellsworth^38^ for details) for representative subsets of the data are 110 m in east‐west direction and 120 m north–south, while the mean value of vertical errors is 162 m.

#### CAT3

This catalogue contains the absolute locations of 440,727 events in the range -1 ≤ M_L_ ≤ 5.58 described in Spallarossa *et al*.^39^ covering the period from 2016-08-24 to 2017-08-31. One entire year of seismic activity reconstructed with the information derived from all the 155 permanent and temporary (stand-alone) stations installed soon after the first (Amatrice) mainshock of the sequence by both INGV mobile network pool, the British Geological Survey and Edinburgh University. Event detection, P- and S-wave arrival times and maximum amplitudes to be used for local magnitude computation, were automatically estimated using a combination of the Complete Automatic Seismic Processor (CASP^40^) and RSNI-Picker2 procedures^41,42^. Arrival time residuals were minimized using the grid search program NonLinLoc^35^ together with a 1D velocity model with homogeneous layers (after De Luca *et al*.^43^) and station corrections calibrated for the area. For each event, location quality was quantified by means of the procedure proposed by Michele *et al*.^44^. It is noteworthy that the CAT3 catalogue includes 30 events with M_L_ > 3.5 missed by the automatic procedure. These events, taken from INGV bulletin manually generated offline^28^ (http://terremoti.ingv.it), have been added by hand to CAT3 and identified by specific identification codes (“ISI00” plus INGV id).

#### CAT4

This catalogue, described in detail in Waldhauser *et al*.^29^, contains 390,334 events that were relocated by applying the double-difference algorithm HypoDD^36^ to the CAT3 catalog^39^. In addition, for the CAT3 phase picks, cross-correlation derived differential travel times were measured for all event pairs with correlated seismograms at common stations using procedures and parameters similar to the ones described in Waldhauser and Schaff^45^. The same 1D velocity model^34^ as in CAT3 was used. CAT4 consists of hypocenters with the smallest relative location errors, on the order of a few tens of meters or better (see Fig. 4). Thus, it can be considered the most enhanced one in terms of location resolution and the ability to image finest-scale fault geometry and fault zone structures. For inclusiveness, being this a catalogue composed by relocated events, we associated M_W_ from Malagnini and Munafò^46^ to the M_L_.

#### CAT5

With 900,050 events found between 2016-08-15 and 2017-08-15, CAT5 is described in detail by Tan *et al*.^30^. This catalog has the lowest minimum magnitude of completeness. Magnitudes range from −2.6 ≤ M_L_ ≤ 6.1, with local magnitude computed using the calibration derived by Di Bona^47^ specifically for the Italian region. The deep neural network PhaseNet picker^14^ was used to detect earthquakes and measuring P- and S-waves arrival times at same 155 stations used to generate CAT3 and CAT4. The association of phase picks to individual events employs the Rapid Earthquake Association and Location (REAL) package^48^. Starting from the 1D velocity model proposed by Chiaraluce *et al*.^24^, the authors used the Velest code^49^ on a subset of newly detected 5,000 events, to estimate a new 1D optimal P- and S-wave velocity model with station corrections. Preliminary absolute location of all events was then computed with the HypoInverse software^50^. Finally, events with at least 4 P-wave picks and 7 picks in total were relocated using the HypoDD code^38^, achieving errors on the order of several tens of meters (see Fig. 4).

## Data Records

The presented dataset^51^ of six catalogues is available at the repository of the British Geological Survey: 10.5285/5afccfe5-142e-4e93-a6cc-55216fa1db06. The content of each catalogue is described below.

### Header of CAT0

**Id1, Date, Time, Lat, Lon, Depth, Errh, Errv, Gap, Rms, Nphs, Mpi, ML, Mw, Md, ML-MED **where:

- **Id1** is INGV event ID
- **Date** is the date of the event in the format yyyy:mm:dd
- **Time** is the origin time in the format hh:mm:ss.sss
- **Lat** is the latitude in decimal degrees (°)
- **Lon** is the longitude in decimal degrees (°)
- **Depth** is the hypocentral depth in kilometres (km)
- **Errh** is the horizontal error in kilometres (km), computed by using the covariance matrix
- **Errv** is the vertical error in kilometres (km), computed by using the covariance matrix
- **Gap** is the maximum azimuth gap in degrees between stations used for location, expressed in decimal degrees (°)
- **Rms** is the root-mean-square of residuals at maximum likelihood or expectation hypocentre, expressed in seconds (s)
- **Nphs** is the number of readings used for location
- **Mpi** is the preferred magnitude as released by INGV.
- **ML** is the local magnitude
- **Mw** is the TDMT moment magnitude from Scognamiglio^51^ (http://terremoti.ingv.it/tdmt).
- **Md** is the duration magnitude.
- **ML-MED** is the automatic magnitude.

### Header of CAT1

**Id1, Date, Time, Lat, Lon, Depth, Errh, Errv, Gap, Rms, Nphs, Mpi, ML, Mw, Md, Mw-M, Strike, Dip, Rake **where:

- **Id1** is INGV event ID
- **Date** is the date of the event in the format yyyy:mm:dd
- **Time** is the origin time in the format hh:mm:ss.sss
- **Lat** is the latitude in decimal degrees (°)
- **Lon** is the longitude in decimal degrees (°)
- **Depth** is the hypocentral depth in kilometres (km)
- **Errh** is the horizontal error in kilometres (km), computed by using the covariance matrix
- **Errv** is the vertical error in kilometres (km), computed by using the covariance matrix
- **Gap** is the maximum azimuth gap in degrees between stations used for location, expressed in decimal degrees (°)
- **Rms** is the root-mean-square of residuals at maximum likelihood or expectation hypocentre, expressed in seconds (s)
- **Nphs** is the number of readings used for location
- **Mpi** is the preferred magnitude as released by INGV. Usually, this is a Mw, if available
- **ML** is the local magnitude of INGV
- **Mw** is the TDMT moment magnitude from Scognamiglio^52^ (http://terremoti.ingv.it/tdmt).
- **Md** is INGV duration magnitude
- **Mw-M** is the moment magnitude retrieved by Malagnini and Munafò^46^ (hereinafter MM18)
- **Strike** is the strike of the focal mechanism (MM18) expressed in decimal degrees (°)
- **Dip** is the dip of the focal mechanism (MM18) expressed in decimal degrees (°)
- **Rake** is the rake of the focal mechanism (MM18), expressed in decimal degrees (°)

### Header of CAT2

**Id1, Date, Time, Lat, Lon, Depth, Errh, Errv, Gap, Rms, Nphs, Mpi, ML, Mw, Md, Mw-M, Strike, Dip, Rake**

the same of CAT1 with the following exceptions:

- **Errh** that is the mean horizontal error in kilometres (km), retrieved from the full covariance matrix computed by using subsets of the catalogue on which we run the Singular Value Decomposition method (SVD; see Waldhauser & Ellsworth^38^).
- **Errv** is the vertical error in kilometres (km), retrieved from the full covariance matrix computed by using subsets of the catalogue on which we run the Singular Value Decomposition method.

### Header of CAT3

**Id1, Id3, Id4, Date, Time, Lat, Lon, Depth, Errh, Errv, Gap, Rms, Nphs, Qual, Class, ML_s, Std_ML_s, Mpi, Mw-R, Mw-M, Strike, Dip, Rake **where:

- **Id1** is INGV event ID
- **Id3** is Spallarossa *et al*.^39^ reference ID
- **Id4** is CAT4^29^ event ID
- **Date** is the date of the event in the format yyyy:mm:dd
- **Time** is the origin time in the format hh:mm:ss.sss
- **Lat** is the latitude in decimal degrees (°)
- **Lon** is the longitude in decimal degrees (°)
- **Depth** is the hypocentral depth in kilometres (km)
- **Errh** is the horizontal error in kilometres (km), computed by using the covariance matrix
- **Errv** is the vertical error in kilometres (km), computed by using the covariance matrix
- **Gap** is the maximum azimuth gap in degrees between stations used for location, expressed in decimal degrees (°)
- **Rms** is the root-mean-square of residuals at maximum likelihood or expectation hypocentre, expressed in seconds (s)
- **Nphs** is the number of readings used for location
- **Qual** is the numeric quality factor: 0 (best quality) < qf <⥶   1 (worst quality). For details see Spallarossa *et al*.^39^ and Michele *et al*.^44^.
- **Class** is the quality class: A (0–0.25); B (0.25–0.5); C (0.5–0.75); D (0.75–1).
- **ML_s** is the local magnitude computed by Spallarossa. For 30 subsequently added events with M ≥ 3.5 that were originally missing (identified by an ID starting with ‘ISI’) we report INGV’s ML.
- **Std_ML_s** is the standard deviation of local magnitude
- **Mpi** is the preferred magnitude as released by INGV
- **Mw-R** is the moment magnitude retrieved by bilinear regressions (from MM18).
- **Mw-M** is the MM18 moment magnitude
- **Strike** is the strike of the focal mechanism (MM18), expressed in decimal degrees (°)
- **Dip** is the dip of the focal mechanism (MM18), expressed in decimal degrees (°)
- **Rake** is the rake of the focal mechanism (MM18), expressed in decimal degrees (°)

### Header of CAT4

**Id4, Date, Time, Lat, Lon, Depth, EH1, EH2, EZ, AZ, ML_s, Mw-M **where:

- **Id4** is Waldhauser *et al*.^29^ event ID
- **Date** is the date of the event in the format yyyy:mm:dd
- **Time** is the origin time in the format hh:mm:ss.sss
- **Lat** is the latitude in decimal degrees (°)
- **Lon** is the longitude in decimal degrees (°)
- **Depth** is the hypocentral depth in kilometres (km)
- **EH1** is the horizontal projection of the major axis in kilometres (km) of the 95% relative location error ellipses derived from bootstrap analysis. (−9 if not available).
- **EH2** is the horizontal projection of the minor axis in kilometres (km) of the 95% relative location error ellipses derived from bootstrap analysis. (−9 if not available).
- **EZ** is the vertical relative location error in kilometres (km) at the 95% confidence level derived from bootstrap analysis. (−9 if not available).
- **AZ** is the azimuth taken from North, in degrees (°) of the horizontal, 95% relative location error ellipses derived from bootstrap analysis. (−9 if not available).
- **ML_s** is the local magnitude computed by Spallarossa *et al*.^39^
- **Mw-M** is the MM18 moment magnitude

### Header of CAT5

**Id5, Date, Time, Lat, Lon, Depth, EH1, EH2, EZ, AZ, ML-N, ML-mean, ML-median, Std-ML, Mw-REGRE, Split **where:

- **Id5** is Tan *et al*.^30^ event ID
- **Date** is the date of the event in the format yyyy:mm:dd
- **Time** is the origin time in the format hh:mm:ss.sss
- **Lat** is the latitude in decimal degrees (°)
- **Lon** is the longitude in decimal degrees (°)
- **Depth** is the hypocentral depth in kilometres (km)
- **EH1** is the horizontal projection of the major axis in kilometres (km) of the 95% relative location error ellipses derived from bootstrap analysis.
- **EH2** is the horizontal projection of the minor axis in kilometres (km) of the 95% relative location error ellipses derived from bootstrap analysis.
- **EZ** is the vertical relative location error in kilometres (km) at the 95% confidence level derived from bootstrap analysis.
- **AZ** is the azimuth in degrees (°) of the horizontal, 95% relative location error ellipses derived from bootstrap analysis.
- **ML_N** is the number of stations used for the ml computation
- **ML**_**mean** is the mean value of ML
- **ML**_**median** is the median value of ML
- **Std-ML**_std is the standard deviation of ML
- **Mw-REGRE** is converted from ML-median using the modified Grünthal *et al*.^53^ scaling relation for Europe built to convert ML to MW. The relation is MW = 0.0376ML2 + 0.646 ML + 0.817, with the constant adjusted through calibration using ~500 events with Mw estimated from regional waveform fitting^54^.
- **Split** is equal to 1 for split events, otherwise is 0

## Technical Validation

Figure 4 compares the distributions of location uncertainty and quality parameters of the six catalogues. The two rows group the distributions according to the estimation method used to obtain them, i.e., absolute (CAT0,1,3) and relative (CAT2,4,5) location errors. Note that CAT0 has a different (and overly optimistic way) to compute errors compared to CAT1 and CAT3. CAT1 improved the locations of CAT0 events in terms of error, robustness, and reliability of the errors. CAT2 further improved the location error albeit reporting only an average value among all events (see header of CAT2). Since CAT3 contains more events than CAT1 (especially of smaller magnitude), the relative number of events with small horizontal error is considerably smaller than for CAT1.

Figure 5 compares the catalogues in terms of their magnitude frequency distribution (MFD). It illustrates the wider range of magnitude covered by CAT3–5 as compared to CAT0–2. However, one must be aware that the local magnitude, M_L_, below about 2–4 is subjected to a scaling break relatively to the moment magnitude, M_w_, as outlined by, for instance, Munafò *et al*.^55^ and Deichmann^56^, which manifests itself in a departure from an exponential-like Gutenberg–Richter relation (e.g., Herrmann & Marzocchi^18^). A conversion of M_L_ into M_w_ as in CAT3 and CAT5 using regressions is a possible remedy and leads to a steeper MFD (see grey curve in Fig. 5). The figure also reflects the effects of magnitude binning used in each catalogue (only CAT1 and CAT2 use a 0.1 binning, whereas the others have a 0.01 binning).

## Data Availability

For generating the catalogues, the IpoP code^31,32^, the Complete Automatic Seismic Processor (CASP^40^) and RSNI-Picker2^41,42^ are available upon request. All of the other codes are all open access: NonLinLoc software^35^ used for CAT1 and CAT3; HypoDD^36,38^ for CAT2, CAT4 and CAT5; PhaseNet picker^14^, (REAL) package^48^, Velest code^49^ and HypoInverse software^50^ used for generating the dataset of CAT5. The performed processing (Table 1, Figs. 3, 4, and 6) are common statistical representations of the data and do not require custom codes; M_c_^Lilliefors^ was calculated with the Python class of Herrmann and Marzocchi^57^. The *Generic Mapping Tools* (www.soest.hawaii.edu/gmt) were used for creating Fig. 1, the Python graphing library *plotly* (www.plotly.com/python) for creating Figs. 2–5, and Matlab (www.mathworks.com) for creating Fig. 6.
